# Characterizing brain states with Granger causality

**DOI:** 10.1186/1471-2202-14-S1-P17

**Published:** 2013-07-08

**Authors:** Adam B Barrett, Lionel Barnett, Paul Chorley, Andrea Pigorini, Lino Nobili, Melanie Boly, Marie-Aurelie Bruno, Quentin Noirhomme, Steven Laureys, Marcello Massimini, Anil K Seth

**Affiliations:** 1 Sackler Centre for Consciousness Science, Dept. of Informatics, University of Sussex, Brighton, BN1 9QJ, UK; 2 Dept. of Clinical Sciences, University of Milan, Milan, 20157, Italy; 3 Centre of Epilepsy Surgery "C. Munari", Niguarda Hospital, Milan, 20162, Italy; 4 Cyclotron Research Centre, Dept. of Neurology, University of Liege, Liege, B30-4031, Belgium

## 

A key step toward understanding how the brain works is the reliable characterization of directed functional (i.e., causal) connectivity between brain regions, generally during various states of wake and sleep, as well as during task performance. Here we perform a critical analysis of the potential of Granger causality (GC) [1] to confront this challenge. We describe new methodology for rigorously applying GC to steady-state data, validated against analytical calculations, and for the first time, simulated spiking neuron local field potential data. We apply our methods to scalp and intracranial electroencephalographic (EEG) recordings.

The concept of GC is to quantify the extent to which the past of one signal Y predicts the future of another signal X. For 'pairwise' GC, one compares predictions based on the past of just × and on the past of both × and Y. For 'conditional' GC, one quantifies the extent to which the past of Y assists in predicting the future of × beyond the extent to which the pasts of × and all other variables excluding Y predict the future of X. These approaches lead to distinct functional connectivity maps. The conditional approach potentially describes better how each system component processes and communicates *distinct *information; however it is practically more awkward because it requires estimating more parameters.

We review the elegant mathematical properties of the linear autoregressive approach to GC that make it an attractive connectivity measure, e.g. its links to information theory and its insensitivity to amplification level [2,3]. We then discuss challenges in the application to electrophysiology data, such as non-stationarity and statistical bias. We present ways to overcome these challenges, involving data segmentation and permutation analysis [4], and modifications to the basic linear autoregression model. We validate our methods in two ways: first using simulated data from models for which true GC values can be analytically derived; second using simulated local field potential data from a large-scale spiking neuron simulator. The latter allows us to confirm, for the first time, that GC analysis can indeed correctly reproduce the underlying causal structure in realistic neural dynamics. We compare and contrast the ability of pairwise and conditional GC approaches to detect significant changes in functional connectivity between wake, sleep and anaesthesia states across recorded scalp and intracranial EEG variables. Analyses are performed in both the time and frequency domains, and we make use of the first ever code for computing conditional GC in the frequency domain. As predicted, pairwise GC is more sensitive to changes in brain state than full conditional GC; however conditional GC performed on just a few simultaneous variables at once can also give meaningful results.

In summary, we demonstrate rigorous methodology and new code for GC analysis of steady-state data, and illustrate the utility of GC in exposing the functional neural interactions underlying different brain states.
